# Epidemiological and virological findings during multiple outbreaks of equine influenza in South America in 2012

**DOI:** 10.1111/irv.12349

**Published:** 2015-12-11

**Authors:** Cecilia Olguin Perglione, Sarah Gildea, Agustina Rimondi, Samuel Miño, Aldana Vissani, Mariano Carossino, Ann Cullinane, Maria Barrandeguy

**Affiliations:** ^1^ CICVyAINTAInstituto de VirologíaBuenos AiresHurlinghamArgentina; ^2^ Virology UnitThe Irish Equine CentreJohnstownNaasCo. KildareIreland; ^3^ Cátedra de Enfermedades InfecciosasEscuela de VeterinariaUniversidad del SalvadorBuenos AiresArgentina; ^4^ Maxwell H. Gluck Equine Research CenterDepartment of Veterinary ScienceUniversity of KentuckyLexingtonKYUSA

**Keywords:** Argentina, equine influenza, Florida clade 1

## Abstract

**Background:**

In 2012, equine influenza (EI) virus was confirmed as the cause of outbreaks of respiratory disease in horses throughout South America. In Uruguay and Argentina, hundreds of vaccinated thoroughbred horses in training and racing facilities were clinically affected.

**Objective:**

To characterise the EI viruses detected during the outbreak in Uruguay and Argentina.

**Methods:**

Virus was detected in nasopharyngeal swabs by a pan‐reactive influenza type A real‐time RT‐PCR. The nucleotide sequence of the HA1 gene was determined and analysed phylogenetically using mega 5 software. Amino acid sequences alignments were constructed and virus was antigenically characterised with specific ferret antisera. Paired serum samples were tested by haemagglutination inhibition and single radial haemolysis.

**Results:**

The diagnosis of EIV was confirmed by real‐time RT‐PCR, virus isolation and serological testing. The phylogenetic analysis of HA1 gene sequences of 18 EI viruses indicated that all of them belong to clade 1 of the Florida sublineage of the American lineage and are closely related to viruses isolated in the United States in 2012. The HA1 of viruses identified in horses in racing facilities in Maroñas, Uruguay, and in Palermo, Argentina, displayed 100% amino acid sequence identity and were identical to that of a virus isolated in Dubai in 2012, from vaccinated endurance horses recently imported from Uruguay.

**Conclusions:**

The surveillance data reported illustrate the international spread of EI viruses and support the recommendations of the OIE expert surveillance panel to include viruses of the Florida sublineage in vaccines.

## Introduction

Equine influenza (EI) is one of the most economically important respiratory diseases of horses, due to its contagious nature and rapid spread among susceptible horses.^1^, ^2^, ^3^ Equine influenza virus (EIV) is endemic in the European and American continents, and large outbreaks of the disease are often associated with the congregation of horses for equestrian events and competition.^3^, ^4^ Two subtypes of influenza A virus, H7N7 and H3N8, have been associated with this disease in horses.^5^, ^6^ Equine influenza viruses currently circulating in horses belong to the H3N8 subtype and are responsible for widespread outbreaks of disease in both vaccinated and unvaccinated horses.^2^, ^7^ Phylogenetic analysis of the haemagglutinin (HA) gene revealed that equine H3N8 viruses diverged during the mid‐1980s and early 1990s into two distinct evolutionary lineages namely the Eurasian and the American lineages.^8^ Viruses of the Eurasian lineage have not been isolated in recent years.^9^ The American lineage has persisted and diverged into the South America, Kentucky and Florida sublineages.^10^ Subsequent evolution within the Florida sublineage has resulted in the emergence of two distinct clades, designated as Florida clade 1 and Florida clade 2.^11^, ^12^ Florida clade 1 viruses are endemic in the USA but have also caused outbreaks in South Africa,^13^ Japan,^14^ Australia 15 and Europe.^11^, ^16^, ^17^, ^18^, ^19^ Florida clade 2 viruses have predominated in Europe but have also been the cause of major outbreaks of EI in China,^20^ Mongolia 21 and India.^22^

Occasional outbreaks of EI have been reported in South America. In Chile, the first outbreak of EI was reported in 1963,^23^ that is the same year, a H3N8 virus was isolated for the first time from horses in the USA.^6^ H3N8 viruses re‐emerged in Chile in 1985, 1992 and 2006.^24^, ^25^, ^26^ Outbreaks due to H3N8 viruses were recorded in Argentina from 1985 to 2005.^26^ Genetic characterisation of viruses from these outbreaks indicated that they belonged within the South American sublineage of the American lineage.^8^, ^10^, ^12^, ^26^ In January 2012, EIV was confirmed as the cause of outbreaks of respiratory disease in Chile and immediately reported to the World Organisation for Animal Health (OIE). Equine influenza outbreaks were subsequently reported in Brazil, Uruguay and Argentina (http://www.oie.int/wahis_2/public/wahid.php/Wahidhome/Home).

Vaccination is key to the control of EI and many countries such as Argentina implement mandatory vaccination programmes for highly mobile horses. Most major thoroughbred racing authorities have a mandatory vaccination policy to minimise the economic impact of EI outbreaks. Antigenic drift compromises vaccine effectiveness as the ability of vaccinal antibodies to recognise the field virus and protect against clinical disease and virus shedding is diminished.^17^, ^27^ Phylogenetic and antigenic analysis of EI field outbreaks worldwide are reviewed annually by the OIE expert surveillance panel (ESP) to improve vaccine effectiveness by ensuring vaccines contain epidemiologically relevant strains. The ESP recommends that the vaccines contain viruses from both clades of the Florida sublineage of the American lineage. The aim of this study was to characterise the EI virus detected during the multifocal occurrence of disease in Uruguay and Argentina during 2012 and to compare it to recommended vaccine strains. At the time of the outbreak, the majority of the vaccines on the Uruguay and Argentine market contained viruses of the American lineage that predated the emergence of the Florida sublineage.

## Materials and methods

### Sample collection

Nasopharyngeal swabs in viral transport medium and whole blood samples from acutely affected horses were collected in Maroñas (*n = *10), Palermo (*n = *5), La Plata (*n = *5), San Isidro (*n = *4), San Antonio de Areco (*n = *6) and Esquel (*n = *10). In addition, whole blood samples from horses in Maroñas, Palermo, La Plata and San Isidro were obtained during the convalescent period of the disease.

### Influenza virus detection

A pan‐reactive influenza type A real‐time RT‐PCR (qRT‐PCR) targeting the matrix gene was performed for the detection of EIV.^28^ The detection limit of this assay (10^1^ EID_50%_–10^1,5^ EID_50%_) and the *C*_t_ (threshold cycle) value to consider a sample as EIV positive (*C*_t_ value <36) was established on the base of a standard curve generated from serial 10‐fold dilutions of the 10^8^ EID_50%_EIV (data not shown).

### Sequencing and phylogenetic analysis of the HA1 gene

The HA1 gene (1009 bp) from 18 EI qRT‐PCR‐positive nasopharyngeal swabs, including those from Maroñas (*n *=* *3), Palermo (*n *=* *3), La Plata (*n *=* *4), San Isidro (*n *=* *3), San Antonio de Areco (*n *=* *3) and Esquel (*n *=* *2), was amplified by the One‐Step RT‐PCR kit (Qiagen, Westburg, Leusden, the Netherlands) using the primers described by Gildea *et al*.^29^ The PCR products were purified and sequenced by the ‘Unidad de Genómica, Instituto de Biotecnología, INTA, Hurlingham'. The HA1 nucleotide sequence was edited with the bioedit software 7.0.9.0 Sequence Alignment Editor.^30^ Multiple nucleotide and amino acid sequences alignments were constructed in clustal_x v.2 and subsequently edited in mega 5.^31^, ^32^ Phylogenetic analysis using mega 5 software was performed with the HA1 gene sequences determined and selected sequences from the NCBI GenBank database, which are shown in Table S1 of the Supporting Information. Genetic distances were calculated using the Kimura two‐parameter as a model of nucleotide substitution. This was determined as the most appropriate evolutionary model of nucleotide substitution for the data according to modeltest. A phylogenetic tree was constructed using the maximum‐likelihood method with 1000 bootstrap replicates.

### Equine influenza virus isolation

Virus isolation was attempted in 10‐day‐old embryonated hens' eggs. Briefly, 0·2 ml of each swab was inoculated into the allantoic cavity and the eggs were incubated at 34 ± 1°C. After incubation for 72 hours, eggs were chilled at 4°C overnight and the allantoic fluid harvested and tested for haemagglutination activity.^33^ Negative samples were passaged up to three times in embryonated hens' eggs.

### Equine influenza virus antigenic characterisation

Representative EIV isolates from Palermo (A/eq/Palermo/E‐2345‐1/12), La Plata (A/eq/La Plata/E‐2346‐5/12) and San Isidro (A/eq/San Isidro/E‐2357/12) were characterised by the haemagglutination inhibition test (HI) with clade‐specific ferret antisera. Briefly, 4 HA units of each virus was tested with serial dilutions of ferret antisera against A/eq/South Africa/4/03 and A/eq/Donegal/09 (representative strains of the clade 1 Florida sublineage) and A/eq/Meath/07 and A/eq/Kildare/12 (representative strains of the clade 2 Florida sublineage). Geometric mean titres were calculated for three HI tests for each combination.

### Equine influenza antibody detection and quantification

Serum samples obtained from EI affected horses during the acute and convalescent period were tested by HI^33^ against H3N8 representative strains of different EIV lineages, including A/eq/Argentina/93 (American lineage, South American sublineage), A/eq/South Africa/4/03 (American lineage, Florida sublineage clade 1), A/eq/Meath/07 (American lineage, Florida sublineage clade 2), A/eq/Kildare/89 (Eurasian lineage) and A/eq/Argentina/E‐2345‐1/12 which was isolated during the outbreak. Seroconversion between the acute and convalescent serum samples was defined as a fourfold or greater increase in the HI antibody titre.^33^ In addition, antibodies against A/eq/Meath/07 and A/eq/South Africa/04/03, representatives of clade 1 and clade 2 of the Florida sublineage of the American lineage, respectively, were measured in 16 paired serum samples by single radial haemolysis test (SRH).^33^ A seroconversion by SRH was defined as an increase in the area of haemolysis of 25 mm^2^ or more between the acute and convalescent serum samples.^34^

## Results

### Chronological description of the disease events

Early in February 2012, several horses suffering an acute respiratory disease were observed by private veterinarians in Maroñas thoroughbred racing and training facilities, in Montevideo, Uruguay. The official notification of this occurrence to the Uruguay National Animal Health Authorities was made on the 10th March 2012. Sanitary measures were imposed at both national and international levels, to control the spread of the infection.

Clinical signs were characterised by pyrexia, coughing and nasal discharge which was initially serous but quickly became mucopurulent. Young horses (2–3 year old) were most severely affected. The morbidity was approximately 40% in a population of 1700 thoroughbred horses. In Uruguay, racetracks require vaccination certificate for racing. Thus, all the affected horses had been vaccinated at least once.

Two months later, on July 8th, horses exhibiting pyrexia, nasal discharge and cough were observed in the Palermo and La Plata racetracks, which are located in Buenos Aires (BA) city and 60 km from BA, respectively. On July 10th, similar clinical signs were observed among horses in the San Isidro racecourse, located in the BA suburban area. The disease affected approximately 40%, 70% and 10% of a total population of 850, 1700 and 2200 horses in Palermo, La Plata and San Isidro racecourses, respectively. As EI vaccination for movement of horses is mandatory in Argentina, this EI outbreak occurred in a regularly vaccinated population of horses.

**Figure 1:**
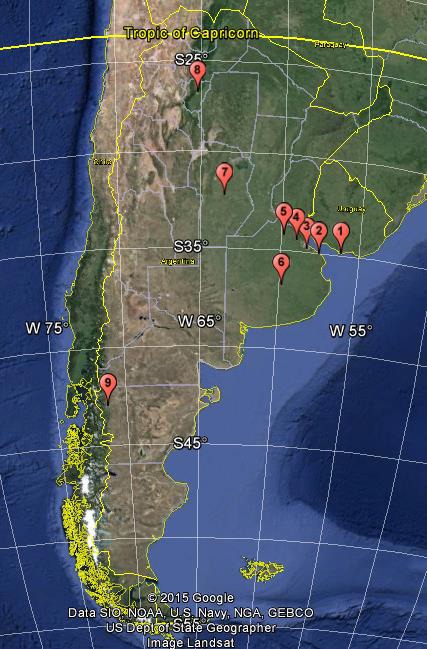
Geographical distribution of premises affected during equine influenza outbreaks in Uruguay and Argentina in 2012. (1) Maroñas, (2) La Plata, (3) Palermo, (4) San Isidro, (5) San Antonio de Areco, (6) Azul, (7) Córdoba, (8) Tucuman and (9) Esquel.

The disease was also confirmed among 80 horses on a farm in San Antonio de Areco (100 km north from BA). Some of these horses had returned from the racetracks in BA. Late in July, the National Animal Health Authorities (SENASA) reported clinical signs consistent with EI in the Azul (300 km south from BA) and Tucuman (1000 km north‐west from BA) racetracks, as well as in a jumping club in Cordoba (500 km north‐west from BA). On 27th November, the disease was confirmed among horses in Esquel, in the province of Chubut (1000 km south from BA), where 25 of 29 horses were affected. The geographical distribution, type of affected premises and vaccination status of the horses are summarised in Figure 1 and Table 1.

**Table 1:** A summary of the geographical distribution, type of affected premises and vaccination status of the affected horses identified by INTA during the influenza outbreaks in Uruguay and Argentina in 2012

Outbreak	Date of diagnosis	Location	Type of premises	Method of detection	Number of horses on premises	Percentage described as clinically affected	Reported vaccination status
1	30th March 2012	Montevideo, Uruguay	Racing and training facilities	qRT‐PCR Serology	1700	40	Vaccinated
2	9th July 2012	La Plata, Buenos Aires, Argentina	Racing and training facilities	qRT‐PCR VI Serology	1700	70	Vaccinated
3	9th July 2012	Buenos Aires city, Argentina	Racing and training facilities	qRT‐PCR VI Serology	850	40	Vaccinated
4	11th July 2012	San Isidro, Buenos Aires. Argentina	Racing and training facilities	qRT‐PCR VI Serology	2200	10	Vaccinated
5	17th July 2012	San Antonio de Areco, Buenos Aires, Argentina	Breeding farm. (Training)	qRT‐PCR VI	80	4	Vaccinated
6	27th November 2012	Esquel, Chubut, Argentina	Show horses	qRT‐PCR	29	86	Unvaccinated

### Virological studies

Equine influenza virus was detected by real‐time RT‐PCR in 27 of 40 nasopharyngeal swabs obtained from affected horses from Maroñas (*n = *8), Palermo (*n = *5), La Plata (*n = *5), San Isidro (*n *=* *4), San Antonio de Areco (*n *=* *3) and Esquel (*n *=* *2). All the positive field samples gave *C*_t_ values between 18·75 and 34·31. No other viruses (equine herpes virus EHV1/EHV4 and equine arteritis virus) were detected by PCR, RT‐PCR or virus isolation (results not shown).

### Phylogenetic analysis of the HA1 gene

**Figure 2:**
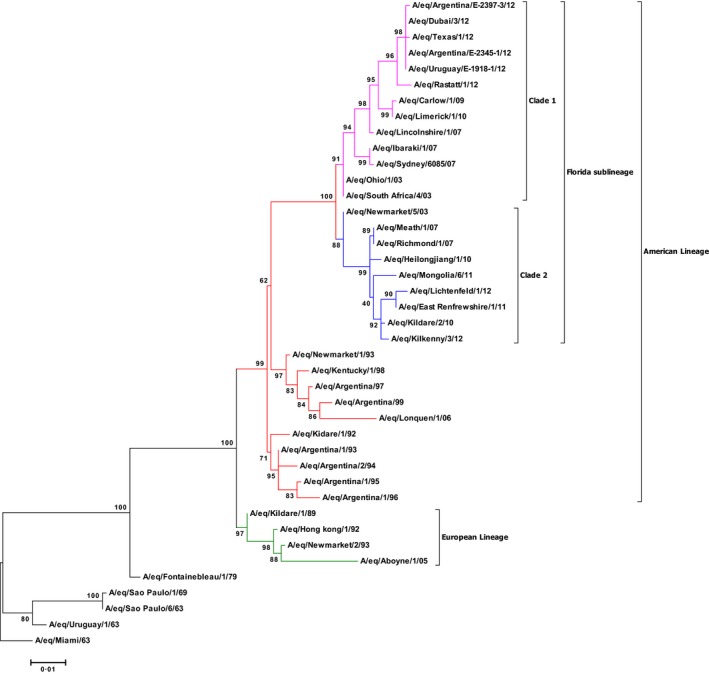
Phylogenetic tree of H3N8 HA1 nucleotide sequence. Bootstrap values obtained after 1000 replicates as shown at the major nodes. Phylogenetic tree is rooted to A/eq/Miami/63. Accession numbers for the genes reported in the manuscript are listed in Supplementary Information Table S1. Branch colours: Black = Pre‐divergent; Green = European lineage; Red = American lineage; Pink = American lineage Florida sublineage clade 1; Blue = American lineage Florida sublineage clade 2.

The phylogenetic analysis of HA1 gene sequences of 18 EI viruses detected in South America indicated that all of them belong to clade 1 of the Florida sublineage of the American lineage (Figure 2) and are closely related to viruses isolated in the United States in 2012.

The viruses identified in Maroñas and Palermo displayed 100% nucleotide identity. Viruses identified in San Isidro, San Antonio de Areco, Esquel and La Plata had 100% HA1 nucleotide identity to each other but had two nucleotide substitutions (G33A and A282G) compared to viruses from Maroñas and Palermo. Viruses with both HA1 nucleotide sequences were identified circulating at La Plata racecourse.

To facilitate the HA1 genome segment analysis, A/eq/Maroñas/E‐1918‐1/2012 (submitted as A/eq/Uruguay/E‐1918‐1/2012 to the NCBI database) and A/eq/Palermo/E‐2345‐1/2012 (submitted as A/eq/Argentina/E‐2345‐1/2012 to the NCBI database) were selected as representative strains with G33 and A282. A/eq/Areco/E‐2397‐3/2012 (submitted as A/eq/Argentina/E‐2397‐3/2012 to the NCBI database) was selected as a representative strain with A33 and G282 (Figure 2).

### Amino acid alignment

**Figure 3:**
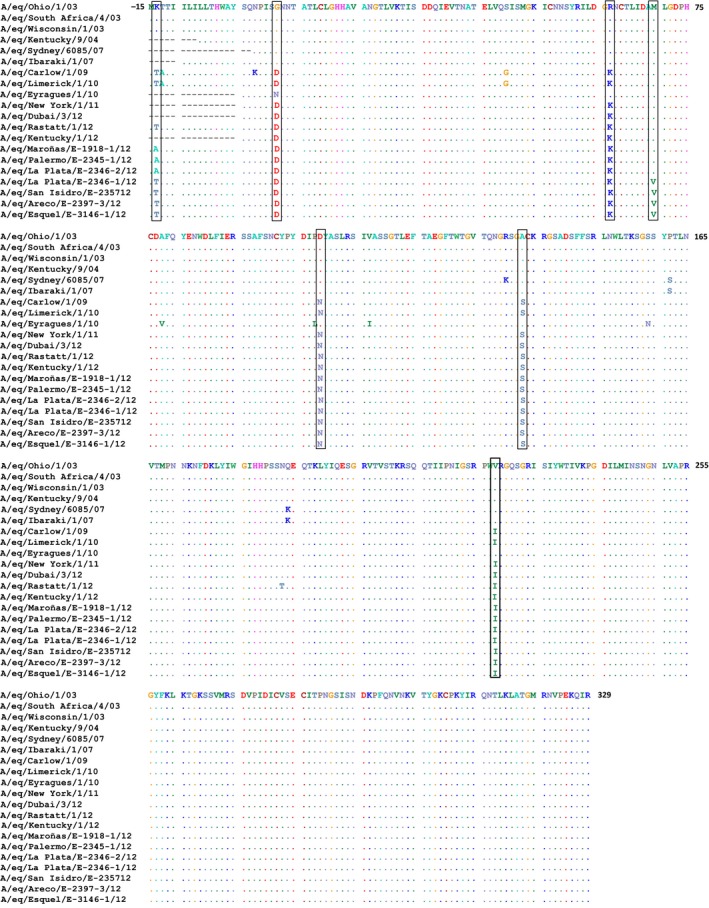
Alignment of HA1 amino acid sequences.

The HA1 gene amino acid sequence of EIV identified in Uruguay and Argentina was aligned with A/eq/Ohio/01/2003, a representative virus of the clade 1 of the Florida sublineage and the amino acid changes are summarised in Figure 3. EIV strains circulating in South America in 2012 were identified as having the characteristic amino acid substitution in the putative antigenic site at position 159 that is likely to define the phenotype of the clade 1 viruses. Furthermore, all Uruguayan and Argentinian 2012 strains showed five amino acid substitutions in comparison with the clade 1 reference strains: G7D, R62K, D104N, A138S and V223I as observed in viruses isolated in Europe and North America.^11^, ^18^, ^19^, ^29^ An additional amino acid substitution (M70V), consequence of the single‐nucleotide change in position 282, was observed when comparing viruses identified in San Isidro (A/eq/San Isidro/E‐2357/12), San Antonio de Areco (A/eq/Areco/E‐2397/12), Esquel (A/eq/Esquel/E‐3146‐1/12) and La Plata (A/eq/La Plata/E‐2346‐1/12) with Florida clade 1 reference strains.^11^, ^18^, ^19^, ^29^ All viruses identified in South America 2012 had an additional substitution at the predicted peptide signal sequence, K‐14T or K‐14A, when compared with the Florida clade 1 reference strain. The substitution K‐14T occurred in A/eq/San Isidro/E‐2357/2012, A/eq/Areco/E‐2397‐3/2012, A/eq/Esquel/E‐3146‐1/2012 and A/eq/La Plata/E‐2346‐1/2012 and in other strains recently circulating in Europe.^11^, ^18^, ^19^, ^29^ The substitution K‐14A, derived from the nucleotide change in the position 33 (A33G), was present in A/eq/Maroñas/E‐1918‐1/2012, A/eq/Palermo/E‐2345‐1/2012 and A/eq/La Plata/E‐2346‐2/2012 and to the authors knowledge has not previously been reported.

### Equine influenza virus isolation

As summarised in Table 1, EIV was successfully isolated from 12 nasopharyngeal swabs collected from horses in Palermo (*n = *5), La Plata (*n = *5), San Isidro (*n = *1) and San Antonio de Areco (*n = *1), but could not be isolated after three passages in embryonated hens' eggs from any of the 10 nasopharyngeal swabs obtained from affected horses in Maroñas (Uruguay).

### Equine influenza virus antigenic characterisation

As expected from their genetic characterisation, ferret antisera against clade 1 viruses recognised the Argentinian isolates to a similar level as their homologous strains, that is an eightfold difference to their titre against clade 2 viruses. In contrast, ferret antisera against the clade 2 viruses gave a lower titre against the Argentinian viruses than against homologous viruses. These titres were similar to those observed with the clade 1 reference virus A/eq/South Africa/4/03 (Table 2).

**Table 2:**
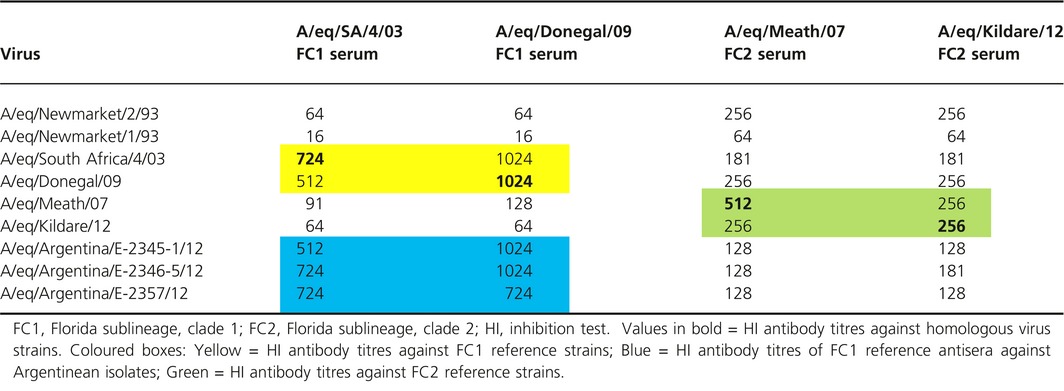
Antigenic characterisation of Argentinian equine influenza virus isolates by HI assay using ferret antisera. Geometric mean titres were calculated for three HI tests for each combination

### Serology

The HI antibody titres against H3N8 and H7N7 in acute and convalescent serum samples are summarised in Table 3. All of the horses (*n = *10) sampled during the EI outbreak in Uruguay were H3N8 seropositive by HI at the time of the initial sampling and only two of them demonstrated an increase in antibody titre 4 weeks later. During the EI outbreak in Argentina, only four of 14 horses tested seropositive in the acute phase of the disease. Convalescent samples were only available for 13 of the 14 horses and all 13 exhibited a significant increase in HI antibody titre during the convalescent period.

**Table 3:**
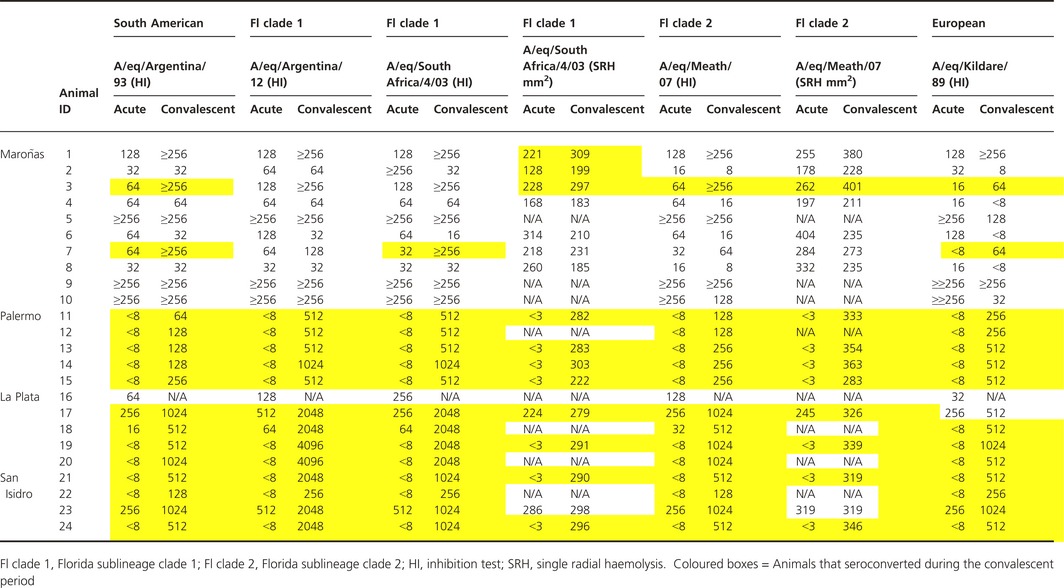
Haemagglutination inhibition and single radial haemolysis results for horses clinically affected in Maroñas, Palermo, La Plata and San Isidro

The SRH antibody titres against A/eq/Meath/07 and A/eq/South Africa/04/03 were consistent with the HI antibody titres in that seven horses tested from Uruguay were seropositive for H3N8 on initial sampling (Table 3). Three of these seven horses seroconverted by SRH during the convalescent period. In Argentina, two of the nine horses tested by SRH were H3N8 seropositive on initial sampling. With the exception of one of these horses, all seroconverted during the convalescent period.

## Discussion

Equine influenza virus infection was the cause of a major outbreak of respiratory disease in thoroughbred horses stabled in Maroñas training and racing facilities in Uruguay, during March and April 2012. Subsequently, from July to November 2012, EIV infection was diagnosed among horses in eight different establishments located in five provinces in Argentina. Six of the affected premises were thoroughbred racing yards, and the other two were facilities for jumping horses and show horses. The movement of horses among thoroughbred training and racing centres is usually very intensive, thus facilitating the spread of the EIV infection throughout the country. No epidemiological link between the thoroughbred yards and the two non‐thoroughbred premises was identified. Equine influenza outbreaks in Brazil were notified to the OIE early in 2012 (http://www.oie.int/wahis_2/public/wahid.php/Wahidhome/Home) and in mid‐March, the Brazilian press reported that 50% of the horses had been withdrawn from races in Porto Alegre and Curitiba racetracks due to an acute respiratory disease (http://www.gripenet.pt/pt/news/2012/03/11/newsletter_2011_17_03/). Horses from these regions had been moved to Uruguay for racing purposes and may have introduced EIV into the racehorse population there. Horses from Uruguay frequently race in Argentina and could have been the source of virus to Argentinian racetracks. Moreover, 3 of 18 endurance horses, which were exported from Uruguay to Dubai on 30th May 2012, exhibited fever and respiratory clinical signs 6 days post‐arrival at quarantine. Equine influenza virus was isolated from nasopharyngeal swabs collected from these horses and genetically characterised.^19^ Our study provides molecular support for the hypothesis that these horses were infected prior to export as the HA1 gene of the virus detected in Maroñas had 100% amino acid sequence identity to the virus isolated in the Dubai quarantine facility. The imported horses had received a primary course of two doses of vaccine in accordance with the manufacturer's recommendations.^19^ The occurrence of EI in these horses is notable, as it illustrates the risk of global virus spread through international movement of vaccinated horses. The role of subclinically infected horses in the international spread of EIV has been highlighted in recent years as has the significant economic losses involved following the introduction of EI into a naïve population.^13^, ^35^, ^36^, ^37^

Equine influenza virus was diagnosed during the outbreaks in Uruguay and Argentina using real‐time RT‐PCR and confirmed by virus isolation and/or HA1 gene nucleotide sequencing. In addition, serological testing confirmed recent exposure to virus in affected horses from both countries. A significant rise in H3N8 antibody titres between paired serum samples was detected by HI and/or SRH. Virus was also isolated from nasopharyngeal secretions collected from horses in Argentina. No virus was isolated from horses in Uruguay. The unsuccessful attempt to isolate the virus from the affected horses in Maroñas may be due to the delay in collection of nasopharyngeal swabs as suggested by the high level of antibody titres found in 80% of horses. However, information relating to the date of last vaccination of the horses was not available. As EI vaccination certification is required to race at Maroñas, it is possible that the horses were seropositive at initial sampling because of recent vaccination and not because of timing of sampling relative to seroconversion due to the outbreak. In contrast, the majority of horses from Argentina were seronegative when nasopharyngeal swabs were obtained. A delay in veterinary intervention and sampling of affected horses has previously been identified as a contributing factor to the rate of diagnosis and therefore virus isolation during recent outbreaks of EI in Europe.^16^, ^17^ Furthermore, our findings of EI real‐time RT‐PCR‐positive nasal swabs but no infective virus are similar to those obtained by Read *et al*.^38^ during the EI outbreak in Australia. In their study, the majority of 36 naturally infected horses tested by positive by real‐time RT‐PCR from the first to the tenth day after clinical signs were evident. However, a minority tested positive for up to 34 days after the onset of clinical signs. In contrast, virus was only isolated from nasal swabs for 6–7 days after clinical signs were first observed.^38^ EIV strains that previously circulated among horses in Argentina, in the 1990s, group within the South American sub‐lineage of the American lineage^8^, ^10^, ^12^ as does the virus that caused the 2006 outbreak in Chile.^26^ However, the molecular and antigenic characterisation of the viruses identified in Uruguay and Argentina in 2012 indicates that they belong to clade 1 of the Florida sublineage of the American lineage and were closely related to viruses circulating in the United States (USA) prior to the outbreak.^19^ In fact, the amino acid sequence of the HA1 gene of the viruses detected in Maroñas and Palermo was identical to that of viruses detected in New York in 2011 and Kentucky in 2012. Outbreaks of EI result in economic losses due to disruption of training programmes, restriction of horse movement and cancellation of equestrian events.^2^ The 2012 EI outbreak in South America resulted in the withdrawal of several horses from races, temporary cancellation of race meetings and a ban on the movement of horses both at national and international level. Additional veterinary and laboratory costs were also incurred as a result of the monitoring and surveillance required to contain the outbreak. Furthermore, following this outbreak, countries such as the United Arab Emirates and Thailand, requested individual proof of freedom from EI infection before importing horses from Argentina in an effort to minimise the risk associated with a virus incursion. The risk of virus shedding associated with international travel can also be mitigated by vaccination with vaccines containing epidemiologically relevant strains. The surveillance data reported in the current study support the recommendations of the ESP to include viruses from the Florida sublineage of the American lineage in vaccines. Many of the vaccine manufacturers in both Uruguay and Argentina have updated their vaccine strains since the 2012 outbreak.

## Source of funding

The investigations and the laboratory work carried out in the Instituto de Virología were supported by INTA and INTA‐Haras agreement. The laboratory work carried out at the Irish Equine Centre was funded by the Department of Agriculture, Food and the Marine.

## Supporting information


**Table S1** Equine influenza (EI) viruses included in phylogenetic analysis.

## References

[1] Timoney PJ . Equine influenza. Comp Immunol Microbiol Infect Dis 1996; 19:205–211. 8800546 10.1016/0147-9571(96)00006-9

[2] Cullinane A , Newton JR . Equine influenza–a global perspective. Vet Microbiol 2013; 167:205–214. 23680107 10.1016/j.vetmic.2013.03.029

[3] Cowled B , Ward MP , Hamilton S , Garner G . The equine influenza epidemic in Australia: spatial and temporal descriptive analyses of a large propagating epidemic. Prev Vet Med 2009; 92:60–70. 19748691 10.1016/j.prevetmed.2009.08.006

[4] Cullinane A , Elton D , Mumford J . Equine influenza – surveillance and control. Influenza Other Respir Viruses 2010; 4:339–344. 20958927 10.1111/j.1750-2659.2010.00176.x PMC4634605

[5] Sovinova O , Tumova B , Pouska F , Nemec J . Isolation of a virus causing respiratory disease in horses. Acta Virol 1958; 2:52–61. 13533033

[6] Waddell GH , Teigland MB , Sigel MM . A new influenza virus associated with equine respiratory disease. J Am Vet Med Assoc 1963; 143:587–590. 14077956

[7] Daly JM , MacRae S , Newton JR , Wattrang E , Elton DM . Equine influenza: a review of an unpredictable virus. Vet J 2011; 189:7–14. 20685140 10.1016/j.tvjl.2010.06.026

[8] Daly JM , Lai AC , Binns MM , Chambers TM , Barrandeguy M , Mumford JA . Antigenic and genetic evolution of equine H3N8 influenza A viruses. J Gen Virol 1996; 77(Pt 4):661–671. 8627254 10.1099/0022-1317-77-4-661

[9] Bulletin O . OIE Expert Surveillance Panel on Equine Influenza Vaccine Composition. Bulletin. Paris: OIE Headquarters, 2014. 4 March 2014. Report No.

[10] Lai AC , Chambers TM , Holland RE Jr *et al* Diverged evolution of recent equine‐2 influenza (H3N8) viruses in the Western Hemisphere. Arch Virol 2001; 146:1063–1074. 11504416 10.1007/s007050170106

[11] Bryant NA , Rash AS , Russell CA *et al* Antigenic and genetic variations in European and North American equine influenza virus strains (H3N8) isolated from 2006 to 2007. Vet Microbiol 2009; 138:41–52. 19346084 10.1016/j.vetmic.2009.03.004

[12] Lewis NS , Daly JM , Russell CA *et al* Antigenic and genetic evolution of equine influenza A (H3N8) virus from 1968 to 2007. J Virol 2011; 85:12742–12749. 21937642 10.1128/JVI.05319-11 PMC3209411

[13] King EL , Macdonald D . Report of the Board of Inquiry appointed by the Board of the National Horseracing Authority to conduct enquiry into the causes of equine influenza which started in the Western Cape in early December 2003 and spread to the Eastern Cape and Gauteng. Aust Equine Vet 2004; 23:139–142.

[14] Yamanaka T , Niwa H , Tsujimura K , Kondo T , Matsumura T . Epidemic of equine influenza among vaccinated racehorses in Japan in 2007. J Vet Med Sci 2008; 70:623–625. 18628606 10.1292/jvms.70.623

[15] Watson J , Daniels P , Kirkland P , Carroll A , Jeggo M . The 2007 outbreak of equine influenza in Australia: lessons learned for international trade in horses. Rev Sci Tech 2011; 30:87–93. 21809755 10.20506/rst.30.1.2021

[16] Gildea S , Arkins S , Cullinane A . Management and environmental factors involved in equine influenza outbreaks in Ireland 2007–2010. Equine Vet J 2011; 43:608–617. 21496094 10.1111/j.2042-3306.2010.00333.x

[17] Gildea S , Fitzpatrick DA , Cullinane A . Epidemiological and virological investigations of equine influenza outbreaks in Ireland (2010–2012). Influenza Other Respir Viruses 2013; 7(Suppl 4):61–72. 24224821 10.1111/irv.12192 PMC5655889

[18] Bryant NA , Rash AS , Woodward AL *et al* Isolation and characterisation of equine influenza viruses (H3N8) from Europe and North America from 2008 to 2009. Vet Microbiol 2011; 147:19–27. 20580170 10.1016/j.vetmic.2010.05.040

[19] Woodward AL , Rash AS , Blinman D *et al* Development of a surveillance scheme for equine influenza in the UK and characterisation of viruses isolated in Europe, Dubai and the USA from 2010–2012. Vet Microbiol 2014; 169:113–127. 24480583 10.1016/j.vetmic.2013.11.039

[20] Qi T , Guo W , Huang W *et al* Isolation and genetic characterization of H3N8 equine influenza virus from donkeys in China. Vet Microbiol 2010; 144:455–460. 20153940 10.1016/j.vetmic.2010.01.006

[21] Yondon M , Heil GL , Burks JP *et al* Isolation and characterization of H3N8 equine influenza A virus associated with the 2011 epizootic in Mongolia. Influenza Other Respir Viruses 2013; 7:659–665. 23289427 10.1111/irv.12069 PMC3626732

[22] Virmani N , Bera BC , Singh BK *et al* Equine influenza outbreak in India (2008–09): virus isolation, sero‐epidemiology and phylogenetic analysis of HA gene. Vet Microbiol 2010; 143:224–237. 20053509 10.1016/j.vetmic.2009.12.007

[23] Fuschlocher F , Zurita L , Latorre G , Palavicino I , editor. Influenza Equina en la Provincia de Santiago. Valdivia, Chile: V Convencion Nacional de Medicina Veterinaria, 1963.

[24] Berrios P . Equine influenza in Chile (1963–1992): a possible human case. Rev Chilena Infectol 2005; 22:47–50. 15798869 10.4067/s0716-10182005000100006

[25] Celedon MO , de Negri L , Santibanez M , Berrios P . Brote de influenza equine en Chile causado por el subtipo H3N8. Agro‐Ciencia 1992; 8:47–48.

[26] Muller I , Pinto E , Santibanez MC , Celedon MO , Valenzuela PD . Isolation and characterization of the equine influenza virus causing the 2006 outbreak in Chile. Vet Microbiol 2009; 137:172–177. 19179022 10.1016/j.vetmic.2008.12.011

[27] Newton JR , Daly JM , Spencer L , Mumford JA . Description of the outbreak of equine influenza (H3N8) in the United Kingdom in 2003, during which recently vaccinated horses in Newmarket developed respiratory disease. Vet Rec 2006; 158:185–192. 16474051 10.1136/vr.158.6.185

[28] Spackman E , Senne DA , Myers TJ *et al* Development of a real‐time reverse transcriptase PCR assay for type A influenza virus and the avian H5 and H7 hemagglutinin subtypes. J Clin Microbiol 2002; 40:3256–3260. 12202562 10.1128/JCM.40.9.3256-3260.2002 PMC130722

[29] Gildea S , Quinlivan M , Arkins S , Cullinane A . The molecular epidemiology of equine influenza in Ireland from 2007–2010 and its international significance. Equine Vet J 2012; 44:387–392. 21978127 10.1111/j.2042-3306.2011.00472.x

[30] Hall TA . A user friendly biological sequence alignment and analysis program for Windows 95/98/NT. Nucl Acid Symp 1999; 41:95–98.

[31] Larkin MA , Blackshields G , Brown NP *et al* Clustal W and Clustal X version 2.0. Bioinformatics 2007; 23:2947–2948. 17846036 10.1093/bioinformatics/btm404

[32] Tamura K , Peterson D , Peterson N , Stecher G , Nei M , Kumar S . MEGA5: molecular evolutionary genetics analysis using maximum likelihood, evolutionary distance, and maximum parsimony methods. Mol Biol Evol 2011; 28:2731–2739. 21546353 10.1093/molbev/msr121 PMC3203626

[33] OIE Manual of Standards for Diagnostic Tests and Vaccines, Equine Influenza. Paris: OIE, 2012.

[34] Newton JR , Townsend HG , Wood JL , Sinclair R , Hannant D , Mumford JA . Immunity to equine influenza: relationship of vaccine‐induced antibody in young Thoroughbred racehorses to protection against field infection with influenza A/equine‐2 viruses (H3N8). Equine Vet J 2000; 32:65–74. 10661388 10.2746/042516400777612116

[35] Guthrie AJ , Stevens KB , Bosman PP . The circumstances surrounding the outbreak and spread of equine influenza in South Africa. Rev Sci Tech 1999; 18:179–185. 10190213 10.20506/rst.18.1.1155

[36] Powell DG , Watkins KL , Li PH , Shortridge KF . Outbreak of equine influenza among horses in Hong Kong during 1992. Vet Rec 1995; 136:531–536. 7660556 10.1136/vr.136.21.531

[37] Garner MG , Cowled B , East IJ , Moloney BJ , Kung NY . Evaluating the effectiveness of early vaccination in the control and eradication of equine influenza–a modelling approach. Prev Vet Med 2011; 99:15–27. 20236718 10.1016/j.prevetmed.2010.02.007

[38] Read AJ , Arzey KE , Finlaison DS *et al* A prospective longitudinal study of naturally infected horses to evaluate the performance characteristics of rapid diagnostic tests for equine influenza virus. Vet Microbiol 2012; 156:246–255. 22115969 10.1016/j.vetmic.2011.10.031

