# Supplementary material for: Epidemiological and virological findings during multiple outbreaks of equine influenza in South America in 2012
Source: Influenza Other Respir Viruses. 2015 Dec 11;10(1):37–46. doi: 10.1111/irv.12349 (PMC4687505; doi:10.1111/irv.12349)
Supplement: Supplementary file 1 — Table S1 Equine influenza (EI) viruses included in phylogenetic analysis. [file IRV-10-37-s001.docx]

**Supplementary Information**

**Table 1: Equine Influenza (EI) viruses included in phylogenetic analysis**

| **Location** | **Lineage** | **Virus name** | **Accession Number** |
| --- | --- | --- | --- |
| Miami, USA | Predivergent | A/eq/Miami/63 | M29257 |
| Uruguay | Predivergent | A/eq/Uruguay/1/63 | CY032421 |
| Sao Paulo, Brazil | Predivergent | A/eq/Sao Paulo/6/63 | CY032293 |
| Sao Paulo, Brazil | Predivergent | A/eq/Sao Paulo/1/69 | CY032397 |
| Fontainebleau, France | Predivergent | A/eq/Fontainebleau/1/79 | CY032405 |
| Kildare, Ireland | European | A/eq/Kildare/1/89 | JN222941 |
| Hong Kong, China | European | A/eq/Hong Kong/1/92 | L27597 |
| Newmarket, UK | European | A/eq/Newmarket/2/93 | X85089 |
| Aboyne, Scotland | European | A/eq/Aboyne/1/05 | EF541442 |
| Kildare, Ireland | American | A/eq/Kildare/1/92 | [JN084402](http://www.fludb.org/brc/fluSegmentDetails.spg?ncbiGenomicAccession=JN084402&context=1427278591846) |
| Newmarket, UK | American | A/eq/Newmarket/1/93 | X85088 |
| Kentucky, USA | American | A/eq/Kentucky/1/98 | AF197241 |
| Argentina | South American Clade 1 | A/eq/Argentina/1/93 | L39913 |
| Argentina | South American Clade 1 | A/eq/Argentina/2/94 | AF197245 |
| Argentina | South American Clade 1 | A/eq/Argentina/1/95 | AF197244 |
| Argentina | South American Clade 1 | A/eq/Argentina/1/96 | AF197246 |
| Argentina | South American Clade 2 | A/eq/Argentina/ 97 | AY048077 |
| Argentina | South American Clade 2 | A/eq/Argentina/ 99 | AY048081 |
| Lonquen, Chile | South American Clade 2 | A/eq/Lonquen/1/06 | EU926631 |
| Argentina | South American Clade 2 | A/eq/Argentina/E-2345-1/12 | KJ372713 |
| Argentina | South American Clade 2 | A/eq/Argentina/E-2397-3/12 | KJ372714 |
| Uruguay | South American Clade 2 | A/eq/Uruguay/E-1918-1/12 | KJ372712 |
| Ohio, USA | Florida sublineage C1ade 1 | A/eq/Ohio/1/03 | DQ124192 |
| South Africa | Florida sublineage C1ade 1 | A/eq/South Africa/4/03 | GU447312 |
| Rastatt, Germany | Florida sublineage C1ade 1 | A/eq/Rastatt/1/12 | KC584975 |
| Texas, USA | Florida sublineage C1ade 1 | A/eq/Texas/1/12 | KF026410 |
| Dubai, United Arab Emirates | Florida sublineage C1ade 1 | A/eq/Dubai/3/12 | KF026413 |
| Ibaraki, Japan | Florida sublineage C1ade 1 | A/eq/Ibaraki/1/07 | AB360549 |
| Sydney, Australia | Florida sublineage C1ade 1 | A/eq/Sydney/6085/07 | GU045763 |
| Lincolnshire, UK | Florida sublineage C1ade 1 | A/eq/Lincolnshire/1/07 | FJ195398 |
| Carlow, Ireland | Florida sublineage C1ade 1 | A/eq/Carlow/1/09 | JN222939 |
| Limerick, Ireland | Florida sublineage C1ade 1 | A/eq/Limerick/1/10 | JN222940 |
| Newmarket, UK | Florida sublineage C1ade 2 | A/eq/Newmarket/5/03 | FJ375213 |
| Meath, Ireland | Florida sublineage C1ade 2 | A/eq/Meath/1/07 | JN222935 |
| Richmond, UK | Florida sublineage C1ade 2 | A/eq/Richmond/1/07 | FJ195395 |
| Mongolia | Florida sublineage C1ade 2 | A/eq/Mongolia/6/11 | AB745622 |
| Lichtenfeld, Germany | Florida sublineage C1ade 2 | A/eq/Lichtenfeld/1/12 | JX499136 |
| Heilongjiang, China | Florida sublineage C1ade 2 | A/eq/Heilongjiang/1/10 | JQ265982 |
| Kildare, Ireland | Florida sublineage C1ade 2 | A/eq/Kildare/2/10 | KC871537 |
| Kilkenny, Ireland | Florida sublineage C1ade 2 | A/eq/Kilkenny/3/12 | KC871549 |
| East Renfrewshire, UK | Florida sublineage C1ade 2 | A/eq/East Renfrewshire/1/11 | KF049198 |
